# Silencing of FTO inhibits oxidative stress to relieve neuropathic pain by m6A modification of GPR177

**DOI:** 10.1002/iid3.1345

**Published:** 2024-07-18

**Authors:** Li Liu, Mei Liu, Zhiping Song, Huaigen Zhang

**Affiliations:** ^1^ Department of Oncology Jiangxi Provincial People's Hospital Nanchang China; ^2^ Department of Anesthesiology The First Affiliated Hospital of Nanchang University Nanchang China

**Keywords:** FTO, m6A methylation modification, neuropathic pain, oxidative stress, YTHDF2

## Abstract

**Background:**

Neuropathic pain (NP) is a challenging health condition owing to its complex nature and associated multiple etiologies. The occurrence of NP involves the abnormal activity of neurons mediated by oxidative stress (OS). Previous research has demonstrated that m6A methylation plays a role in the regulatory pathway of NP. This study aimed to investigate the specific molecular pathways through which m6A methylation modifiers alleviate NP.

**Methods:**

For this purpose, an NO rat model was developed via spared nerve injury (SNI), followed by quantifying the animal's pain assessment via paw withdrawal threshold (PWT) and paw withdrawal latency (PWL). The OS in SNI rats was evaluated by measuring reactive oxygen species, superoxide dismutase, and catalase (CAT) in spinal cord tissues. Moreover, quantitative‐real‐time polymerase chain reaction and western blot analysis were employed for detecting fat mass and obesity‐associated (FTO) and GPR177 levels, while m6A levels of GPR117 were analyzed via MeRIP.

**Results:**

The results indicated an enhanced OS with highly expressed FTO in spinal cord tissue samples, where knocking down *Fto* effectively relieved NP and OS in SNI rats. Mechanistic investigations revealed that *Fto*‐mediated reduction of *Grp177* m6A modification was involved in the WNT5a/TRPV1 axis‐mediated OS remission of NP. Moreover, in vitro experiment results indicated that YTHDF2 was an important m6A methylated reading protein for this process.

**Conclusions:**

*Fto* silencing leads to increased m6A methylation of *Grp177* through a YTHDF2‐dependent mechanism, resulting in decreased *Grp177* stability and ultimately reducing NP in rats by OS suppression.

## Graphical abstract

A working scheme of the potential mechanism of FTO in neuropathic pain.

## 1 INTRODUCTION

Chronic neuropathic pain (NP) is a common disease associated with sensory nerve injury and characterized by persistent pain without external stimuli^1^, ^2^ and is defined as “pain arising from the actual or potential damage to nonneural tissue and results from the activation of nociceptors” by the International Association for the Study of Pain,^3^ with a 14.6% of incidence reported in 2022.^4^ Distinct symptoms in individuals with NP include diabetic neuropathy, neuropathic back and neck pain, and hereditary or idiopathic neuropathy.^5^ Patients may experience enduring or sporadic burning, stabbing, and squeezing pain, and it can extend to adjacent tissues within the patient's body, seriously affecting their quality of life.^6^ The inadequate comprehension of the pathogenesis makes treating NP a significant clinical challenge. Currently, most drugs used to treat NP often have side effects that restrict the dosage.^7^ It has been found that interaction between the immune and nervous systems plays a vital role in NP, where immune cells are involved in regulating neuroinflammation, further driving the onset, progression, and end of NP.^8^ Therefore, it is imperative to investigate the immune‐related pathogenesis in NP with an aim to explore novel therapeutic targets and treatment strategies.

Oxidative stress (OS) manifests due to an imbalanced production and elimination of reactive oxygen species (ROS) within the body,^9^ where superoxide dismutase (SOD) and catalase (CAT) have been recognized as vital enzyme‐based antioxidant systems.^10^ OS and neuroinflammation are involved in the occurrence and maintenance of NP.^11^ Pathologically, the occurrence of NP is associated with a large number of ROS, which in turn involves mitochondrial dysfunction, glial activation, and inflammatory reactions in patients.^12^ When the typical operation of mitochondria is hindered, a significant quantity of ROS is released, resulting in OS in vivo and due to the release of inflammatory cytokines and OS‐related enzymes by the inflammatory cells driving OS onset. Chronic and consistent cooccurrence of OS and inflammation leads to central sensitization, aggravating NP.^13^ Thus, it is imperative to investigate the regulatory mechanism of OS in NP. The abnormal activity of mature neurons following OS is a key factor that promotes NP progression. The capsaicin receptor (TRPV1) in mature neurons functions in transducing injurious signals and generating pain as a heat‐sensitive ion channel^14^; hence, lower TRPV1 expression can effectively inhibit NP progression.^15^ WNT5a belongs to the Wingless‐type MMTV integration site family,^16^ acting on neurons through the β‐catenin‐independent WNT signaling pathway, and has been reported to be involved in NP development.^17^ Recent research indicates that the activation of TRPV1 by WNT5a is essential for NP formation, which is regulated by GPR177,^18^ which was an intriguing factor in this study since studies on exploring pathways and regulatory mechanisms in this process are scarce.

GPR177 is a member of the G protein‐coupled receptor (GPCR) family,^19^ where all members have similar stereoscopic structures (seven transmembrane alpha helices).^20^ Moreover, GPCR effects on NP progression have recently been identified,^21^ where different GPCRS have been reported to be methylated by m6A,^22^, ^23^ a key RNA modification mechanism, primarily driven by methylated transferases (writers: METTL3, METTL14, WTAP, RBM15, and VIRMA, etc.), demethylases (erasers: *FTO* and ALKBH5, etc.) and RNA binding proteins (readers: YTHDF2, YTHDC1, YTHDF1, YTHDC2, YTHDF3, IGF2BP2, IGF2BP1, and IGF2BP3, etc.).^24^ The m6A methylation is also found to be involved in RNA splicing, maturation, transcription, translation, and stability,^25^ as well as m6A modification, has been identified to regulate various physiological and pathological processes, such as epithelial−mesenchymal transition and OS.^26^, ^27^ However, whether GPR177 can undergo methylation by m6A during NP progression is not yet reported.

This study aimed to investigate the specific molecular pathways through which m6A methylation modifiers alleviate NP and to investigate the involvement of FTO in NP and its control over m6A methylation of *Grp177*.

## 2 MATERIALS AND METHODS

### 2.1 Animals

All male SD rats (weight ≥200 and <250 g) were purchased from the National Rodent Seed Center of China. The rats were housed at 25°C and humidity of 50%−60%, with a 12:12 h light and dark cycle. They had free access to feed and water. The experiment strictly adhered to the Animals (Scientific Procedures) Act 1986. The animal study was approved by the Animal Protection and Utilization Committee of the First Affiliated Hospital of Nanchang University.

### 2.2 Spared nerve injury (SNI)

All rats were divided into eight groups, sham, SNI, SNI + sh‐NC, SNI + sh‐FTO, SNI + sh‐NC + lv‐NC, SNI + sh‐FTO + lv‐NC, SNI + sh‐NC + lv‐GPR177, and SNI + sh‐FTO +lv‐GPR177 groups, with six rats each group. The NP rat model was established using sciatic nerve resection as previously described.^28^, ^29^, ^30^ Briefly, rats were anesthetized with isoflurane (1.5%−2.5%), and a lateral thigh incision was made. The left sciatic nerve and its three peripheral branches were isolated after sciatic nerve resection. A 5‐0 suture was used to ligate the common peroneal and tibial nerves. Then, distal transverse incisions were made at the ligation. All surgical procedures were completed within 20 min, with special care taken to avoid damaging the sural nerve. The rats in the sham group underwent the same sciatic nerve exposure operation as the SNI group except for nerve ligation.

Lentivirus targeting short hairpin RNA *Fto* (sh‐*Fto*, GCTGCACCTACAAGTACTTGA), *Gpr177* overexpressed lentivirus (lv‐*Gpr177*), and their negative control lentivirus (sh‐NC and lv‐NC) were purchased from Obio Technology Corp. One week before model establishment, the PE‐10 catheter was intrathecally implanted in the subarachnoid space of lumbar enlargement in rats. Then, rats were given intrathecal injections of 10 μL lentivirus (1 × 10^8^ TU/mL), followed by injection of 10 μL normal saline to wash the catheter. Behavioral tests were performed at 1, 3, 7, and 14 days after establishing the model. Finally, rats were killed, and the whole L3−L5 spinal cord segments were collected.

### 2.3 Pain behavioral quantification

Paw withdrawal threshold (PWT) and paw withdrawal latency (PWL) were measured to analyze the pain levels of the rats.^31^ Rats were placed in transparent boxes featuring metal mesh floors. On Days 1, 3, 7, and 14 following the SNI surgery, the pressure on the plantar surface of the rat hind paw was measured by a Von Frey electrometer (UGO). For the PWL experiment, the Plantar Test Apparatus/Tail Flick Test Analgesia Meter Apparatus (IITC) was used to obtain PWL values at the same time points post‐SNI surgery. These behaviors were evaluated by two investigators. They did not know which group the rats belonged to and the detailed treatment methods in each group.

### 2.4 ROS measurement

ROS levels were measured using 2′,7′‐dichlorofluorescin diacetate (DCFH‐DA; Beyotime) according to the manufacturer's instructions. Briefly, the spinal cord samples were washed with PBS and ground. The homogenate is filtered through a 45 μm nylon mesh. The single‐cell suspension was collected and incubated with DCFH‐DA at 37°C for 60 min in the dark. The cells were centrifuged at 1000*g* for 10 min and washed with serum‐free medium. Subsequently, fluorescence emission peaks were detected using BD FACSCalibur (BD Biosciences).

### 2.5 SOD measurement

After rat spinal cord samples were collected, sucrose buffers were added to form homogenates. The homogenate was centrifuged. Then the supernatant was collected. WST SOD Assay Kit (NobieRyder) was used to measure SOD values. In short, WST working solution, dilution buffer, enzyme working solution, and supernatant are mixed and incubated together for 20 min. Finally, the HBS‐1096A microplate reader (DeTie Laboratory Equipment) was used to determine OD values at 450 nm.

### 2.6 CAT measurement

This work was conducted according to the study of Asghar et al.^32^ H_2_O_2_ solution (50 mM) was used as the substrate, and the reaction solution was prepared in 50 mm potassium phosphate buffer. The decomposition of H_2_O_2_ by CAT was detected using a UV‐7502CS spectrophotometer (Arda) at 240 nm. The PH of the solution was maintained at 7.0 throughout our experiment.

### 2.7 Cell culture

To investigate the m6A modification of *Grp177* mediated by FTO, in vitro experiments were performed. The rat pheochromocytoma cell line (PC‐12) was purchased from the China Center for Type Culture Collection Kunming Cell Bank and cultured under standard conditions (37°C and 5% CO_2_). PC‐12 cells were cultured in complete DMEM (base DMEM: fetal bovine serum: penicillin/streptomycin = 89:10:1). When the cells grew to about 85% confluence, 0.25% (w/v) trypsin solution was used for cell passage.

### 2.8 Cell transfection

sh‐*Fto*, sh‐*Igf2bp1* (GCGATGAAGGCCATCGAAACT), sh‐*Igf2bp*2 (GGGTAAAGTGGAGTTGCATGG), sh‐IGF2BP3 (GCAGAGGATTCGGAAACTTCA), sh‐*Ythdf1* (GGATGCAGTTCATGACAATGA), sh‐*Ythdf*2 (GGTTCTGCATAGACTGCAGCA), and their negative control (sh‐NC, CAACAAGATGAAGAGCACCAA) were purchased from GenePharma. They were transfected into PC‐12 cells using Lipofectamine 2000 reagent (Invitrogen), according to the manufacturer's instructions.

### 2.9 Quantification of mRNA expression levels by quantitative‐real‐time polymerase chain reaction (qRT‐PCR)

TRIzol reagent (Takara) was used to extract total RNA. Oligo dT primers (Macrogen) and RR047A reverse transcription kit (Takara) were used to prepare cDNA. Quantitative PCR analysis was performed using the Mir‐X miRNA qRT‐PCR TB Green kit (Takara) on the ABI Prism 7700 sequence detection System (Applied Biosystems). All responses were repeated three times. Relative mRNA expression was calculated using the $2^{‐\Delta \Delta C_{t}}$method. Forward and reverse primers related to the study are listed in Table 1. GAPDH acted as the internal control.

**Table 1:** Primers used for qRT‐PCR.

Name	Sequence (5′‐3′)
IGF2BP1‐F	CGGCAACCTCAACGAGAGT
IGF2BP1‐R	GCGTAGCCGGATTTGACCAA
IGF2BP2‐F	TTCTCAGGCCAGACAGATTGA
IGF2BP2‐R	CTCCTTTCCGATGATGGCACC
IGF2BP3‐F	CGCCCCACTTACAATGGGAG
IGF2BP3‐R	CTGCCGTTTCCGAATCCGT
YTHDF1‐F	GAGCAGTTACACTTACCCACC
YTHDF1‐R	TGTTGAGGGAGTCACTGTGAAA
YTHDF2‐F	GAGCAGAGACCAAAAGGTCAAG
YTHDF2‐R	CTGTGGGCTCAAGTAAGGTTC
FTO‐F	CCGTCCTGCGATGATGAAGT
FTO‐R	CCCATGCCGAAATAGGGCTC
GPR177‐F (mature)	CAGTCCAAGTGAACAGTGCC
GPR177‐R (mature)	CTCCTGGGCCTCCTTGCG
GPR177 precursor‐F	TGGAGGTGACAGGAGGGT
GPR177 precursor‐R	ACTGCCGTTGTGGGTGCT
GAPDH‐F	ATTGTTGCCATCAATGACCC
GAPDH‐R	AGTAGAGGCAGGGATGATGT

Abbreviation: qRT‐PCR, quantitative‐real‐time polymerase chain reaction.

### 2.10 RNA sequencing (RNA‐seq)

TRIzol reagent (Takara) was used to extract total RNA, followed by measuring RNA purity and concentration using a cole‐parmer ultrafine spectrophotometer (SPEX CertiPrep). RNAs were fragmented and synthesized with double‐stranded cDNA. The sequencing was conducted using the Illumina HiSeq. 4000 platform (Illumina), while the heatmap was generated using the Origin software (EA).

### 2.11 Stabilization assay

Actinomycin D (2 mg/mL; Merck) was used to treat PC‐12 cells for 0, 2, 4, 8, and 12 h. The mRNA expression of *Grp177* was measured by qRT‐PCR.

### 2.12 Quantification of protein expression levels by western blot analysis

Total protein was extracted using RIPA lysis buffer (Strong) (Maokang Biotechnology). The total protein was transferred to SDS‐polyacrylamide gel and then subjected to constant pressure electrophoresis. The isolated proteins were transferred to the PVDF membrane (Merck). The membrane was incubated with primary antibodies at 4°C for 12 h. Horseradish peroxidase‐conjugated goat anti‐rabbit (ab205718, 1:5000, Abcam) and goat anti‐chicken (ab6877, 1:5000, Abcam) were added for incubation of 2 h. An enhanced chemiluminescence assay system (Millipore) was used to visualize the antigen‐antibody complex. The primary antibodies involved in the article were as follows: FTO antibody (ab126605, 1:2000, Abcam), GPR177 antibody (ab72385, 1:2000, Abcam), WNT5a antibody (ab235966, 1:1000, Abcam), TRPV1 antibody (ab305299, 1:1000, Abcam), and GADPH antibody (ab9485, 1:2500, Abcam).

### 2.13 MeRIP assays

EpiQuik CUT & RUN m6A RNA Enrichment (MeRIP) Kit (GepigenTek) was used to detect the m6A levels in *Grp177*. Protein A magnetic beads (20 μL) were mixed with the m6A antibody at 4°C. The complex was then added to the cell lysate and incubated overnight at 4°C. Finally, qRT‐PCR was used to detect GPR177 expression in immunoprecipitated RNA.

### 2.14 Dual‐luciferase reporter assay

M6A methylation sites in *Grp177* were predicted using the online SRAMP software (http://www.cuilab.cn/sramp). A total of four possible sites were obtained, named sites 1, 2, 3, and 4. To explore which sites can be demethylated by FTO, wild‐type (WT) cDNA of *Grp177* containing site 1, 2, 3, or 4 m6A motif was cloned into the pGL3 vector. A Site‐directed Mutagenesis Kit (Sangon Biotech) was used to replace adenosine (A) in the m6A motif with cytosine (C) to synthesize corresponding mutated (MUT) reporter plasmids. Each WT or MUT reporter plasmid was cotransfected with sh‐NC and sh‐FTO into PC‐12 cells. To explore the binding relationship between GPR177 and YTHDF2, full‐length 3′UTR of *Grp177* was inserted into the pGL3 vector to synthesize WT reporter plasmid and MUT plasmid was also generated. The MT or MUT plasmid was cotransfected with sh‐NC or sh‐YTHDF2 into PC‐12 cells. The luciferase activity was measured using the luciferase reporter assay system (Promega) after 48 h.

### 2.15 Statistical analysis

Statistical analysis was performed using the SPSS 18.0 software. Data were expressed as mean ± standard deviation. Differences between the two groups were compared by unpaired *t*‐test using homoscedastic analysis. If the *p* value of the *F* test <0.1, heteroscedastic analysis was performed to correct *p* value. Differences among multiple groups were compared with one‐way ANOVA followed by Tukey's post hoc test. All experiments were performed in triplicate. The statistical significance level was set at *p* < .05.

## 3 RESULTS

### 3.1 OS response and m6A modification enzyme expression in SNI rats

**Figure 1:**
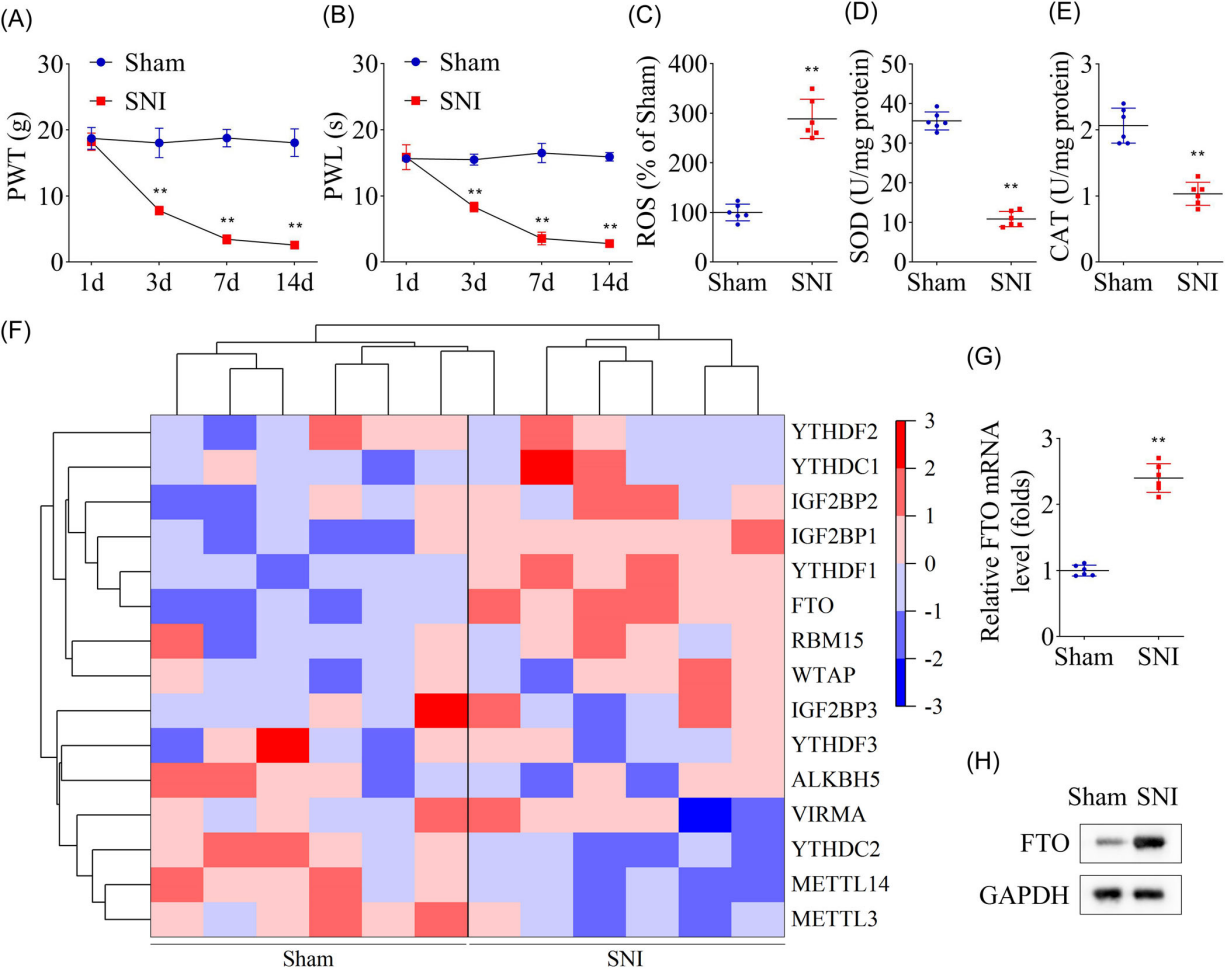
Oxidative stress response and m6A modification enzymes expression in SNI rats. After the SNI model was established for 1, 3, 7, and 14 days, the levels of PWT (A) and PWL (B) in SNI rats. The measurement of ROS (C), SOD (D), and CAT (E) in the spinal cord tissues of SNI rats. (F) Heatmap of relative mRNA expression of m6A modification enzyme in SNI rats. (G) The protein levels of FTO in the spinal cord tissues of rats were detected by western blot analysis. *n* = 6 for each group. Data were analyzed using a *t*‐test. **p < .01. CAT, catalase; PWL, paw withdrawal latency; PWT, paw withdrawal threshold; ROS, reactive oxygen species; SNI, spared nerve injury; SOD, superoxide dismutase.

SNI rat model was established, and NP was determined on 1, 3, 7, and 14 days post‐model establishment, and results showed significantly reduced PWT and PWL values on Day 3, which continued to reduce for 14 days (Figure 1A,B), cementing successful establishment of NP rat model. It was then proceeded by assessing OS effects in SNI rats, where L3–L5 spinal cord tissues were collected post‐behavioral test and subjected to OS evaluation. Results indicated that ROS levels were significantly elevated in SNI rats (Figure 1C); however, SOD and CAT activity was significantly decreased (Figure 1D,E), suggesting SNI‐induced OS response. Moreover, analysis of mRNA levels of m6A methylation‐related genes in rats revealed that *Igf2bp1*, *Ythdf1*, and *Fto* were highly expressed in SNI rats (Figure 1F,G), and FTO protein levels in the spinal cord of SNI rats were also found elevated (Figure 1H). We inferred from the results above that FTO was the primary modifying enzyme regulating OS in NP.

### 3.2 Knockdown of *Fto* inhibited NP in SNI rats

**Figure 2:**
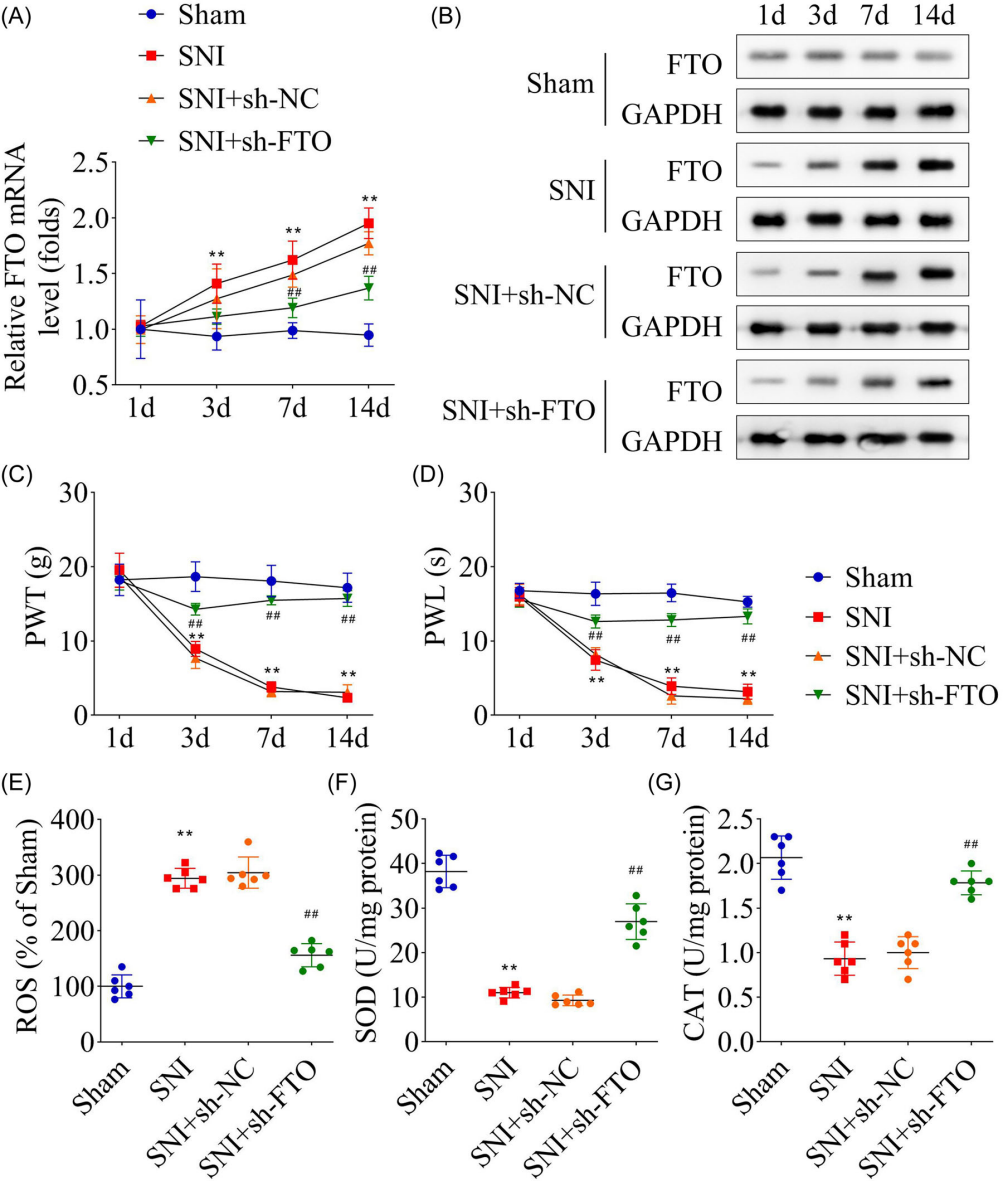
FTO knockdown inhibited NP and OS in SNI rats. After FTO was knocked down and the SNI model was established, FTO mRNA (A) and protein (B) levels in the spinal cord tissues were measured using qRT‐PCR and western blot analysis, respectively. The PWT levels (C) and PWL levels (D) of SNI rats after FTO knockdown after the model was established for 1, 3, 7, and 14 days. ROS (E), SOD (F), and CAT (G) were measured in the spinal cord tissues of SNI rats after FTO knockdown. *n* = 6 for each group. Data were analyzed using one‐way ANOVA. **p < .01, ^##^ *p* < .01. CAT, catalase; NP, neuropathic pain; OS, oxidative stress; PWL, paw withdrawal latency; PWT, paw withdrawal threshold; qRT‐PCR, quantitative‐real‐time polymerase chain reaction; ROS, reactive oxygen species; SNI, spared nerve injury; SOD, superoxide dismutase.

The expressions of *Fto* were knocked down to verify FTO involvement in regulating OS, and results showed that the *Fto* mRNA and protein levels were elevated in SNI rats as a function of time (Figure 2A,B), which reduced with post‐sh‐*Fto* transfection. NP evaluation in SNI rats revealed that PWT and PWL decreased continuously within 14 days, which were reversed by knocking down *Fto* (Figure 2C,D), suggesting *Fto* knockdown effectively attenuated NP in SNI rats. Moreover, ROS detection results showed that *Fto* knockdown inhibited ROS enrichment in the spinal cord of SNI rats (Figure 2E), in addition to decreasing SOD and CAT accumulation (Figure 2F,G). These results collectively indicated that *Fto* could affect NP in rats by regulating the OS process.

### 3.3 m6A methylation modification of *Gpr177* by *Fto*

**Figure 3:**
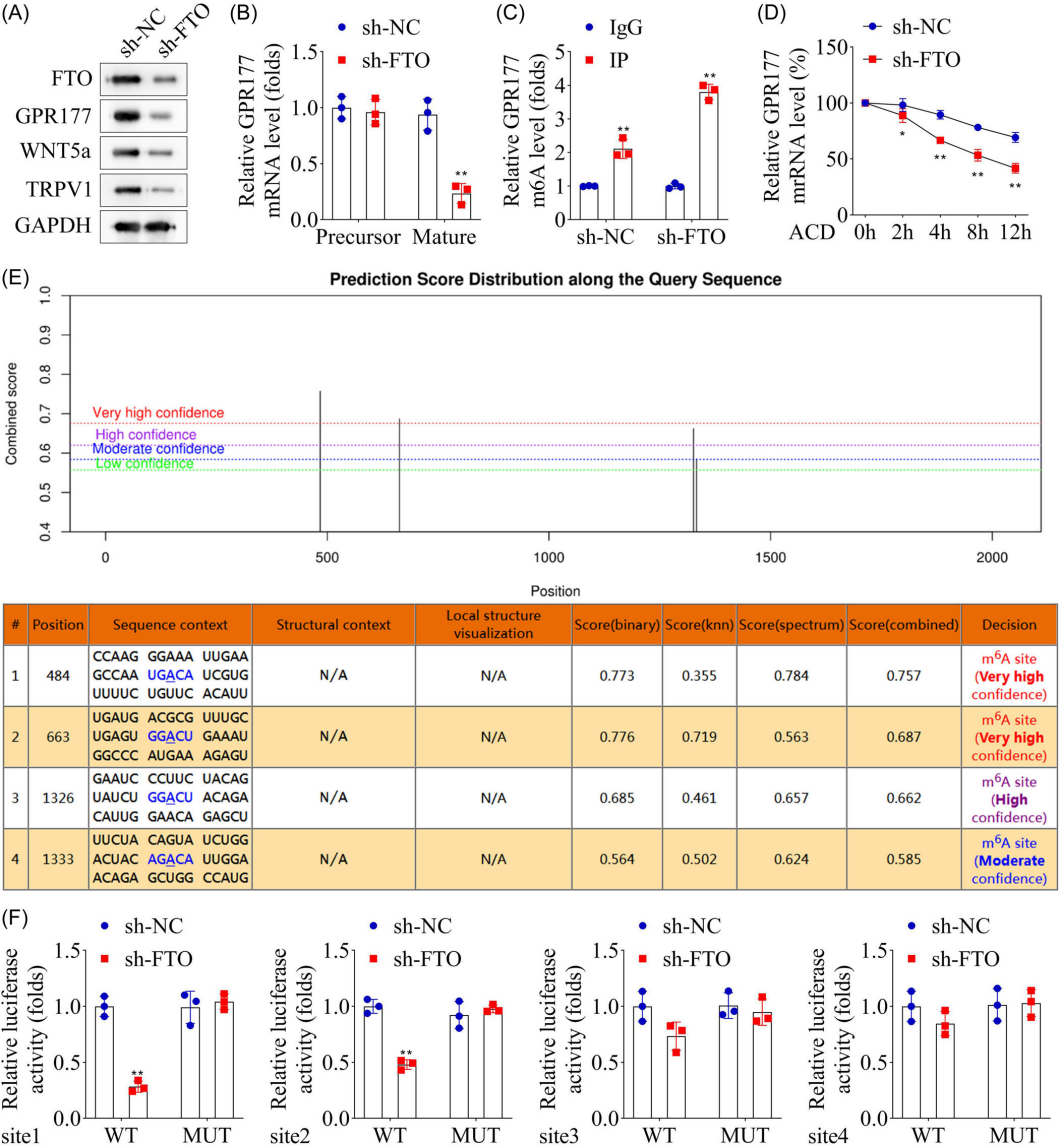
m6A modification of GPR177 by FTO in PC‐12 cells. (A) Western blot analysis was used to detect the protein levels of FTO, GPR177, WNT5a, and TRPV1. (B) mRNA expression levels of GPR177 were detected by qRT‐PCR. (C) MeRIP assays of GPR177 m6A levels after sh‐FTO transfection. (D) MRNA stability of GPR177 after sh‐FTO transfection. (E) The m6A methylation sites in GPR177 were predicted by the SRAMP online analysis software. (F) Dual‐luciferase report assay was used to analyze methylation sites in GPR177 mediated by FTO. *n* = 3 for each group. Data were analyzed using a *t*‐test. *p < .05, **p < .01. qRT‐PCR, quantitative‐real‐time polymerase chain reaction.

Prior research demonstrated that GPR177 in A‐fiber dorsal root ganglion neurons mediates the release of WNT5a into the cerebrospinal fluid, thereby activating TRPV1 to induce a rapid current, suggesting the potential involvement of the GPR177/WNT5a/TRPV1 axis in NP development.^18^ Western blot analysis revealed significantly reduced protein levels of FTO, TRPA1, WNT5A, and GPR177 post‐*Fto* knockdown (Figure 3A). Analysis of *Gpr177* mRNA levels post‐*Fto* knockdown showed that the expressions of *Gpr177* maturation were decreased, as shown in Figure 3B, whereas the *Gpr177* precursor expression remained unaffected, cementing a positive regulatory relationship between *Fto* and *Gpr177*. Moreover, MeRIP assays showed that the m6A methylation modification level of *Gpr177* was significantly increased due to decreased *Fto* expression (Figure 3C), revealing FTO‐mediated m6A modification of *Gpr177*, in addition to reduced *Gpr177* mRNA stability (Figure 3D), cementing the positive regulation and m6A modification effect of FTO on *Gpr177*. The SRAMP online software predicted m6A sites of *Gpr177*, revealing four possible m6A methylation sites (Figure 3E). Synthetic reporter plasmids (WT or MUT) targeting specific sites in *Gpr177* were synthesized to determine which sites can be demethylated by *Fto*, where WT plasmids contained intact m6A sites, whereas the MUT plasmids eliminated the m6A methylation in these sites. Results of the dual luciferase reporting experiment showed that the luciferase activity of the Sites 1 and 2 WT group was significantly decreased post‐*Fto* knockdown, while the MUT group remained unaffected. Moreover, the luciferase activity remained unaffected by *Fto* regardless of whether Sites 3 and 4 were MUT or MUT (Figure 3F), suggesting that FTO can affect *Gpr177* m6A modification at Sites 1 and 2 but not at Sites 3 and 4. These results collectively confirmed the m6A methylation of FTO on GPR177.

### 3.4 The regulation of GPR177 by *FTO* affected NP in SNI rats

**Figure 4:**
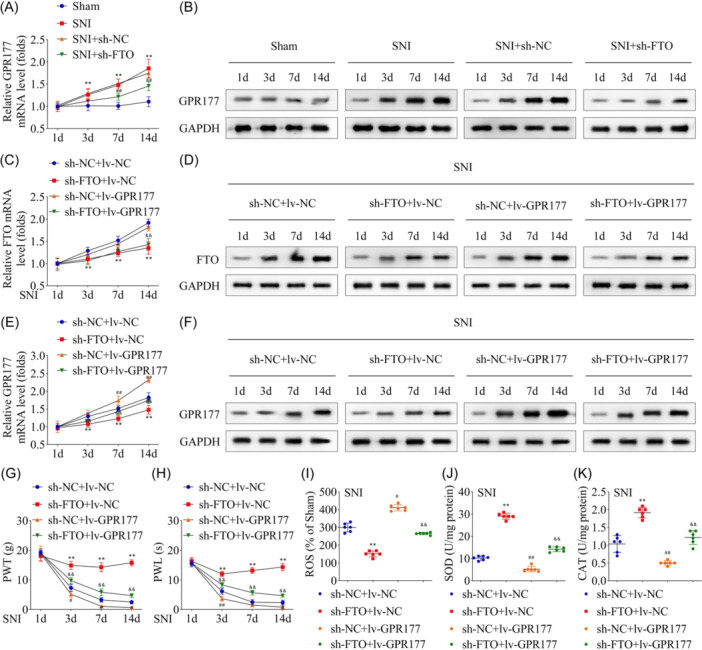
GPR177 reversed the NP and OS in SNI rats mediated by FTO knockdown. After FTO was knocked down and the SNI model was established, GPR177 mRNA (A) and protein (B) levels in the spinal cord tissues were measured using qRT‐PCR and western blot analysis, respectively. After FTO silence and GPR177 overexpression, FTO mRNA (C) and protein (D) levels, and GPR177 mRNA (E) and protein (F) levels were detected in the spinal cord tissues. After FTO silence and GPR177 overexpression, the SNI rat model was established, and PWT (G) and PWL (H) were measured at 1, 3, 7, and 14 days. ROS (I), SOD (J), and CAT (K) were measured in the spinal cord tissues of SNI rats after GPR177 overexpression. *n* = 6 for each group. Data were analyzed using one‐way ANOVA. **p < .01, ^##^ *p* < .01, ^#^ *p* < .05, ^&&^ *p* < .01, ^&^ *p* < .05. CAT, catalase; NP, neuropathic pain; OS, oxidative stress; PWL, paw withdrawal latency; PWT, paw withdrawal threshold; qRT‐PCR, quantitative‐real‐time polymerase chain reaction; ROS, reactive oxygen species; SNI, spared nerve injury; SOD, superoxide dismutase.

m6A modification on *Gpr177* in the onset of NP was confirmed by injecting sh‐*FTO* and lv‐GPR177 into rats, followed by establishing the SNI model. Results showed that GPR177 at mRNA and protein levels were upregulated in SNI rats, which were reversed by knocking down *Fto* (Figure 4A,B). Furthermore, sh‐*Fto* decreased FTO and GPR177 levels, while lv‐GPR77 elevated GRP177 levels but failed to change FTO levels. Similarly, sh‐*Fto*‐induced downregulated FTO expression was reversed by overexpressing *Gpr177* (Figure 4C–F). It was proceeded by determining NP on Day 1, 3, 7, and 14 post‐model establishments, and results showed that PWT and PWL values were significantly increased in the low *Fto* expression group compared to the control (Figure 4G,H), effectively alleviating NP in SNI rats, which were reversed by overexpressing *Gpr177*. Evaluation of OS effects in spinal cord tissue samples of SNI rats post *Gpr177* overexpression revealed reversal of *Fto* knockdown‐induced inhibition of ROS content (Figure 4I). Similarly, *Gpr177* overexpression also significantly inhibited the increased SOD and CAT accumulation levels post‐ *Fto* knockdown (Figure 4J,K).

### 3.5 YTHDF2 recognized *FTO*‐mediated m6A modification of *Gpr177*

**Figure 5:**
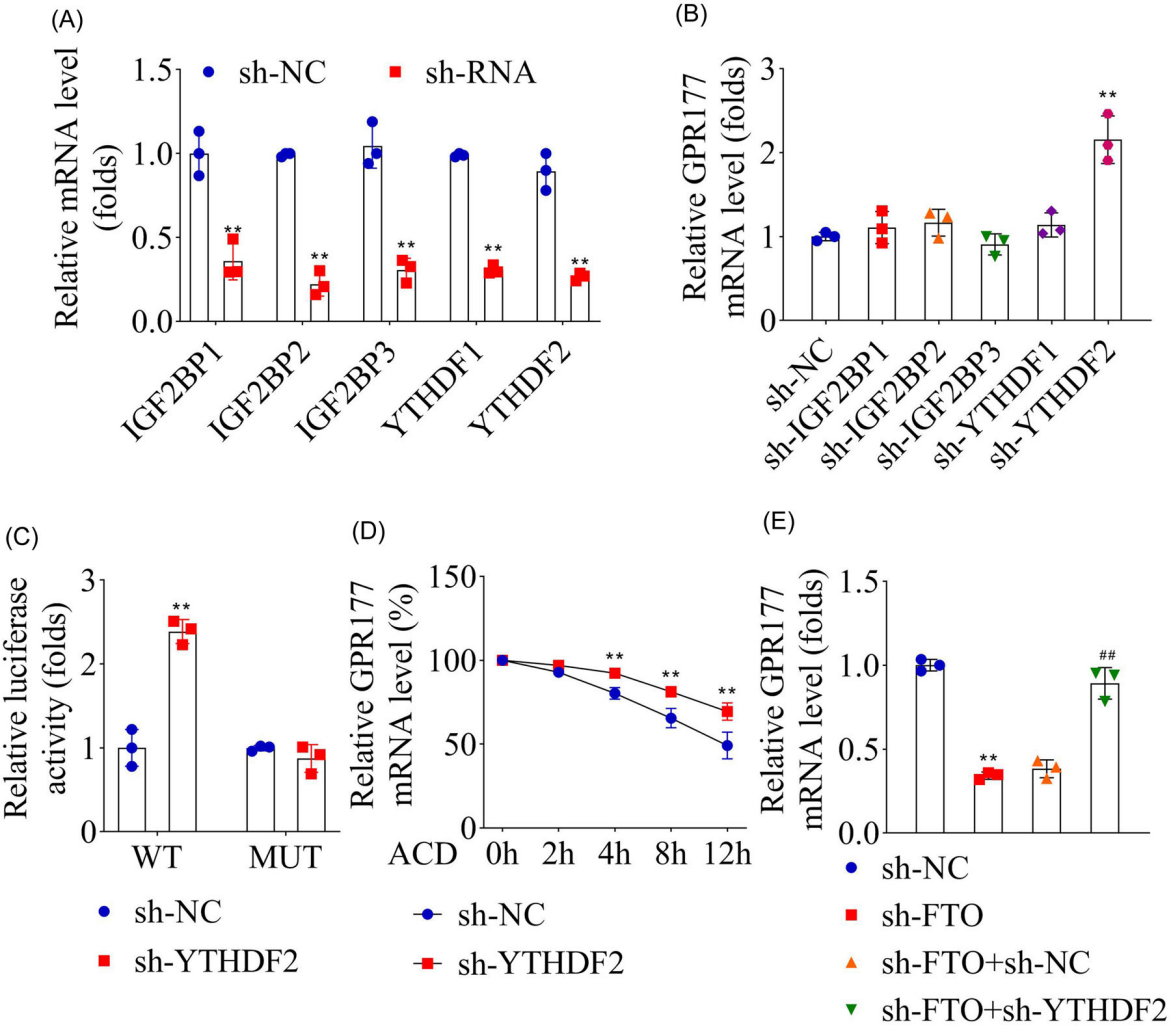
YTHDF2 recognized m6A methylation of GPR177 mediated by FTO in PC‐12 cells. The transfection efficiencies of sh‐IGF2BP1, sh‐IGF2BP2, sh‐IGF2BP3, sh‐YTHDF1, and sh‐YTHDF2 were determined by qRT‐PCR. (B) mRNA expression levels of GPR177 were detected by qRT‐PCR. (C) Dual‐luciferase assay confirmed the binding relationship between YTHDF2 and FTO. (D) The mRNA stability of GPR177 was determined by Actinomycin D assay. (E) mRNA expression levels of GPR177 after sh‐YTHDF2 and sh‐FTO transfection were detected by qRT‐PCR. *n* = 3 for each group. Data were analyzed using *t*‐test (A−D) and one‐way ANOVA (E). **p < .01, ^##^ *p* < .01. qRT‐PCR, quantitative‐real‐time polymerase chain reaction.

To investigate the complete m6A methylation modification pathway, the expression levels of the members of the YTHDF (Ythdf1/2) and IGF2BP (Igf2bp1/2/3) families in PC‐12 cells were reduced. Results of qRT‐PCR indicated significantly decreased mRNA levels of *Ythdf1/2* and *Igf2bp1/2/3* (Figure 5A), indicating successful transfection. Similarly, the mRNA expression level of *Gpr177* after *Ythdf1* and *Igf2bp1/2/3* knocking down did not change, while the mRNA level of *Gpr177* was significantly increased with reduced *Ythdf2* expression (Figure 5B), indicating *Ythdf2* knockdown promoted *Gpr177* enrichment. Hence, it was speculated that YTHDF2 was the key “reader” in the m6A methylation modification of *Gpr177*, which was confirmed by dual luciferase reporting experiment and stability detection. Results showed that the luciferase activity of WT‐GPR177 was significantly increased post‐*Ythdf2* knockdown, but that of MUT‐GPR177 was not regulated by *FTO* (Figure 5C). In addition, when the expression level of *YTHDF2* was decreased, the stability of *Gpr177* mRNA was enhanced (Figure 5D). Finally, *Gpr177* expression regulated by *Fto* and *Ythdf2* was detected, and results showed that *Ythdf2* knockdown reversed the low expression of *Gpr177* caused by *Fto* knockdown (Figure 5E). Hence, it was concluded that YTHDF2 can specifically recognize m6A‐modified *Gpr177* regulated by *Fto*.

## 4 DISCUSSION

**Figure 6:**
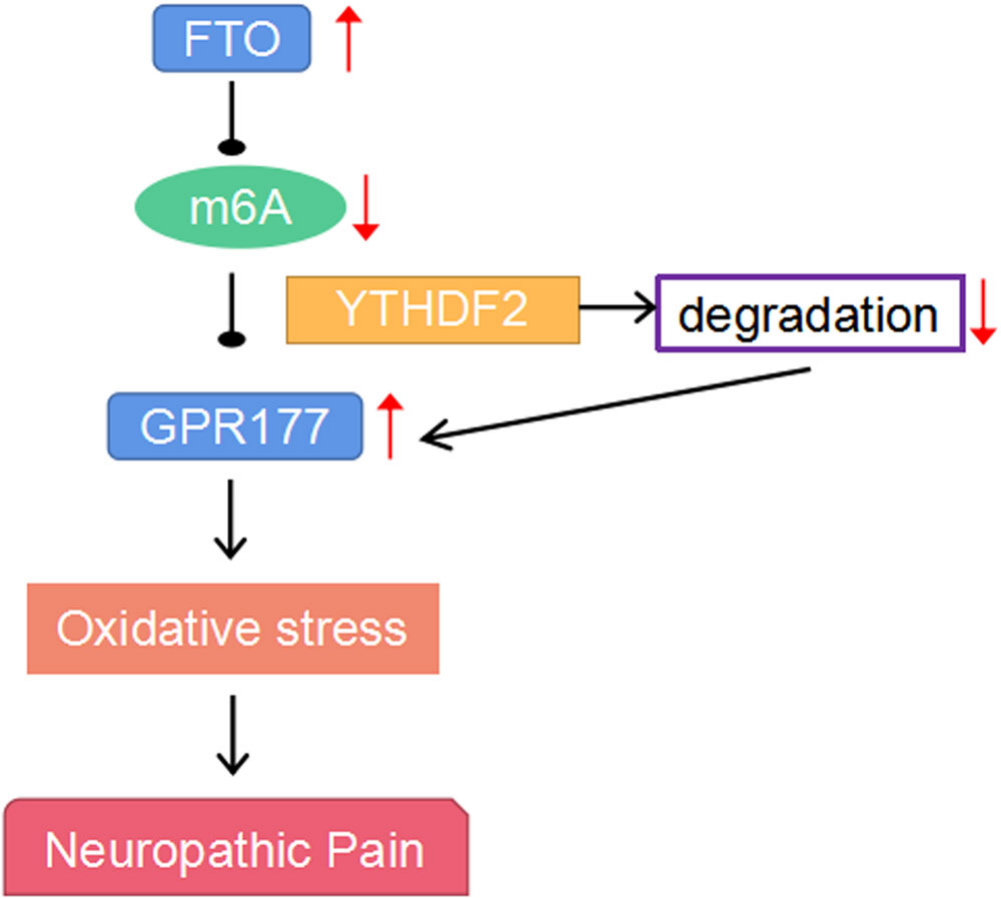
A working scheme of the potential mechanism of FTO in NP. NP, neuropathic pain.

NP is a common chronic disease caused by abnormal neuronal activity resulting in hyperalgesia and abnormal pain.^1^, ^2^, ^3^, ^7^, ^33^ Due to an insufficient understanding of the NP pathogenesis, NP treatment remains a major clinical challenge.^5^, ^6^, ^7^, ^34^ This study aimed to analyze the role of an m6A eraser, *FTO*, in NP progression and investigate the underlying mechanism. Our study results showed that FTO inhibited m6A methylation of *Gpr177*, whose degradation may be suppressed by YTHDF2, leading to GRP177 overexpression, and the FTO/GRP177 axis was involved in NP by regulating OS (Figure 6).

NP is a highly challenging disease to treat and is a multifaceted condition resulting from various diseases or injuries, including traumatic spinal cord injury, persistent diabetes, lumbar disc syndrome, cancer, and HIV infection.^35^, ^36^, ^37^ Moreover, NP progression is regulated by the involvement of immune response since immune cells contribute to pain hypersensitivity or alleviating pain.^8^ An activated immune system releases a variety of cytokines and chemokines that contribute to the development of OS,^38^ which is regarded as vital in the onset and maintenance of NP. Accumulation of excessive ROS induces abnormal activity in mature neurons,^39^ aggravating NP in patients. Moreover, NP increases the enrichment of m6A methylation, where inhibition of m6A methylation has been reported to alleviate NP.^40^ FTO, a m6A eraser, is found to be involved in the onset and maintenance of NP^41^; however, its involvement in NP progression by regulating OS is not widely reported. The FTO was found to be highly expressed in SNI rats, related to OS, where silencing of FTO was found to reduce ROS level, increase SOD and CAT activity, and alleviate NP, suggesting FTO contributes to NP by inducing OS.

FTO functions as a demethylase to facilitate m6A modification of mRNA to promote NP. It was reported that FTO‐mediated m6A modification of G9a translates into NP symptoms^42^ while knocking down FTO was found to relieve NP progression by promoting CXCR3 methylation.^43^ Hence, the possible modification of RNA by FTO was investigated in this study. NP is associated with abnormal neuronal activity often accompanied by abnormal expression of the WNT signaling pathway, transient receptor potential (TRP) family, and other key enzymes. A recent study has shown that GPR177 drives the generation of NP by inducing the activation of TRPV1 via WNT5a mediation.^18^ Our results indicated that FTO knockdown downregulated the GPR177, WNT5a, and TRPV1 protein levels, suggesting that FTO affects the GPR177/WNT5a/TRPV1 axis in NP, with GPR177 as an upstream factor; hence, GPR117 was focused and regulatory effects of FTO on GPR117 were investigated. GPR177 belongs to the highly conserved GPCR protein family, several members of which are methylated by m6A and are involved in disease progression, for example, m6A methylated GPR133 was found to promote lung adenocarcinoma cell proliferation and tumor growth,^44^ while METTL3 was reported to promote m6A modification of GPR166 to stimulate defensin expression, thereby impeding *Escherichia coli* infection.^45^ Nevertheless, whether GPR177 can be modified by m6A methylation remains unclear. Our results showed that *Fto* knockdown decreased mature *Grp177* expression; the precursor expression of GPR177 remained unaffected, suggesting that *Fto* affected *Grp177* expression at the transcriptional level. The m6A methylation is a form of RNA modification occurring at the transcriptional level. Therefore, FTO‐mediated m6A modification in *Grp177* was investigated, and results demonstrated that *Fto* knockdown promoted m6A methylation of *Grp177* and reduced *Grp177* mRNA stability, thereby decreasing *Grp177* mRNA and protein levels. Taken together, the silencing of *Fto* demethylated *Grp177* and inactivated the GPR177/WNT5a/TRPV1 axis, alleviating NP in rats.

YTHDF2 is a widely studied m6A “reader” that can recognize m6A modification sites. A large number of targets for YTHDF2 have been found, most of which are mRNA. It has been reported that YTHDF2 specifically binds to RNAs who containing m6A and then facilitates degradation of target transcripts.^46^ In addition, YTHDF2 has also been reported to regulate pre‐ribosomal RNA (pre‐rRNA) processing.^47^ However, little is known about which “reader” can recognize m6A methylation of *Grp177*. In our study, among these “readers,” only *Ythdf2* knockdown was found to increase *Grp177* expression, while its knocking down enhanced *Grp177* mRNA stability and reversed the *Fto* knockdown induced downregulated *Grp177*, demonstrating that YTHDF2 recognized m6A demethylation of *Grp177* mediated by FTO, while FTO silencing promoted m6A methylation of *Grp177* in a YTHDF2‐dependent manner to inhibit OS in NP.

Nevertheless, there are certain limitations in this study. Inflammation interacting with OS was identified as a pathology of NP, but the roles of proinflammatory and anti‐inflammatory cytokines and the functions of inflammatory cells during the progression of NP have not been clearly elucidated. We only investigated the regulatory impact of FTO on pain and OS in male rats without considering gender variations. Moreover, the SNI model is inadequate in accurately representing the entire chronic NP process. Additionally, since our results investigating the role of YTHDF2 were based on an in vitro model, its role in vivo remains unclear. We aim further to investigate the inflammation response in our future research. Furthermore, additional clinical studies are required to determine if FTO can serve as a therapeutic target for NP.

## 5 CONCLUSION

This study demonstrates that silencing of *Fto* alleviates NP by suppressing OS by downregulating GRP177 expression levels. Mechanically, *Fto* knockdown mediates m6A methylation of *Gpr177*, putatively, in a YTHDF2‐dependent manner and reduces *Gpr177* stability, thereby decreasing GPR177 levels. These findings provide a new potential target for clinical treatment of NP.

## AUTHOR CONTRIBUTIONS

All authors participated in the design, interpretation of the studies, analysis of the data, and review of the manuscript. Li Liu drafted the work and revised it critically for important intellectual content. Mei Liu and Zhiping Song were responsible for the acquisition, analysis, or interpretation of data for the work. Huaigen Zhang made substantial contributions to the conception or design of the work. All authors read and approved the final manuscript.

## CONFLICT OF INTEREST STATEMENT

The authors declare no conflict of interest.

## ETHICS STATEMENT

We strictly followed the Animals (Scientific Procedures) Act 1986 in the animal experiment. All the steps involved in this study were strictly approved by the Animal Protection and Utilization Committee of The First Affiliated Hospital of Nanchang University.

## Data Availability

The data sets used and/or analyzed during the current study are available from the corresponding author upon reasonable request.
